# Clinical and radiographic evaluation of non-surgical therapy with and without ozone gel application in controlled type 2 diabetic patients with periodontitis: a randomized controlled clinical trial

**DOI:** 10.1186/s12903-024-05212-7

**Published:** 2024-11-25

**Authors:** Abeer Abubaker Barahim, Nesma Shemais, Arwa Mousa, Mona Darhous

**Affiliations:** 1 Oral Medicine & Periodontology Department, Faculty of Dentistry, Cairo University, Cairo, Egypt; 2 Oral and Maxillofacial Radiology Department, Faculty of Dentistry, Cairo University, Cairo, Egypt

**Keywords:** Periodontitis, Diabetic patients, Ozone, Scaling Root Planing, Clinical attachment level, Ozone gel

## Abstract

**Background:**

The current study aimed to assess the clinical and radiographic outcomes of the effect of subgingival application of ozonated gel as an adjunct to scaling and root planing (SRP) in diabetic patients with stage III periodontitis.

**Methods:**

Twenty-four patients with type II diabetes mellitus (DM) were randomized into two groups, with 12 patients in each group. Group I served as the intervention group, receiving both SRP and ozone gel application (SRP + Ozone), while Group II served as the control group, undergoing SRP alone. Clinical attachment level was evaluated as primary outcome, with secondary outcomes including probing pocket depth, full mouth plaque score, full mouth bleeding score, dentinal hypersensitivity, radiographic linear defect depth, radiographic defect angle, and periodontal ligament widening space assessed at 3 and 6 months.

**Results:**

The results revealed statistically significant intragroup differences between the two groups (*p* < 0.05). In contrast, intergroup differences revealed no statistically significant difference across the various time intervals (*p* > 0.05). The reduction in PD in the SRP + Ozone group at three months was statistically significant (*p* = 0.04). The SRP + Ozone group showed a significant radiographic improvement compared to the SRP group. The Visual Analogue Scale (VAS) also demonstrated statistically significant differences between the two groups. Glycated hemoglobin (HbA1c) significantly decreased after 6 months, with no significant signifcant differences between groups (*p* > 0.05).

**Conclusions:**

Ozone gel is suggested to be a promising potential natural adjunctive therapy for diabetic patients to enhance periodontal health, with no reported adverse effects.

**Trial registration:**

ID: NCT05538078, Date of Registration: 09/09/2022. (https://register.clinicaltrials.gov/prs/app/action/DownloadReceipt?uid=U0006D54&ts=3&sid=S000CGX4&cx=g1wreh).

## Background

Periodontitis is an inflammatory disease triggered by the accumulation of plaque biofilms [1]**.** It is characterized by tissue loss and alveolar bone resorption. This condition arises from an interaction between the host immune system, pathogenic bacteria, and various established environmental and systemic risk factors [2]. In patients with periodontitis stage III, significant damage to the attachment apparatus occurs, leading to tooth loss if advanced treatment is not provided [3]**.**

Periodontal disease and diabetes mellitus (DM rank among the most prevalent chronic diseases affecting individuals worldwide [4]**.** Bidirectional interactions resulted in a three-fold increase in the likelihood of periodontal disease, while hyperglycemia-induced inflammatory responses impact periodontal tissues [5].

Researchers [6, 7] examined the association between periodontitis, DM, and oxidative stress, indicating that diabetic patients experience increased oxidative stress due to hyperglycemia and AGE formation, contributing to tissue damage. Additionally, high blood sugar complications can directly induce significant production of reactive oxygen species (ROS), resulting in an imbalance between these oxidizing agents, ultimately resulting in oxidative stress and cellular death [6, 7]. Bascones-Martinez et al. [8] investigated the impact of impaired PMN leukocyte function in patients with diabetes, which leads to changes in chemotaxis, adherence, phagocytosis, and bacterial killing. In diabetic patients with periodontitis, there is a significant decrease in the production of anti-inflammatory factors such as interleukin-4, interleukin-10, transforming growth factor-beta, and anti-inflammatory lipid-based mediators, as described by Van Dyke [9]**.** Periodontitis exacerbates insulin resistance by boosting the cytokine-mediated immune response activation [10]. Patients with severe, untreated, periodontal disease exhibited elevated levels of pro-inflammatory mediators, such as IL-1, IL-6, tumor necrosis factor-alpha (TNF-α), and prostaglandin E2 (PGE2), which entered the systemic circulation, triggering an acute-phase response as observed previously [11]**.**

The use of ozone in medical procedures has emerged as a noninvasive therapeutic approach, gaining growing popularity due to its reliable microbiological and metabolic characteristics [12]. Ozone exhibits immunomodulatory, anti-hypoxic, biosynthetic, analgesic, and anti-inflammatory properties, resulting in its extensive utilization in dentistry [13]. It could be further considered a potent oxidizing agent with nonsurgical periodontal treatment (NSPT), potentially decreasing oxidative stress in diabetic patients [3, 14]. This combination has effectively reduced clinical parameters, including PD, CAL, BOP, PI, and GI, as well as microbial counts in patients with periodontitis [13, 15]. Martínez-Sánchez et al. [16] illustrated that using ozone in treating patients with type 2 DM improved antioxidant enzyme activity, directly scavenging free radicals and enhancing glycemic control. This results from reduced hyperglycemia, enhanced insulin sensitivity, and the mitigation of oxidative stress linked to diabetes mellitus and its associated complications.

The combination of ozone therapy with SRP may enhance various periodontal clinical parameters, as demonstrated by different studies [13, 15]**.** However, applying this combination in clinical practice continues to be controversial [17]**.** There was limited evidence regarding the beneficial clinical and radiographic effects of ozone therapy in the management of periodontitis, particularly in patients with type II diabetes mellitus [3]**.** Therefore, the current trial aimed to assess the therapeutic clinical and radiographic outcomes of combining Ozone gel with nonsurgical periodontal treatment in diabetic patients with stage III periodontitis.

## Material and methods

### Study designs and registration

**Fig. 1:**
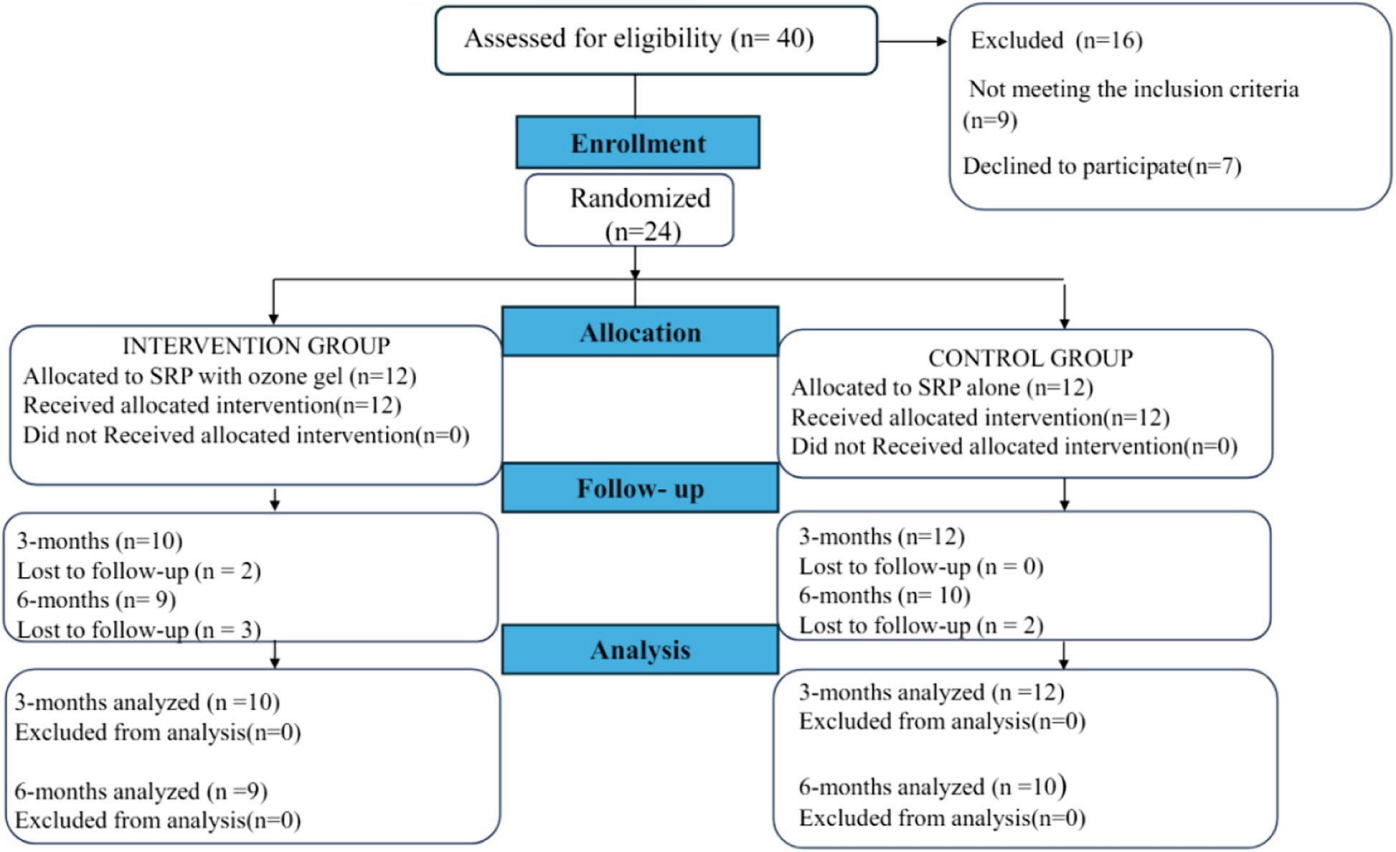
Participants, flow diagram

The current randomized controlled clinical trial enrolled 24 periodontitis patients with controlled type II DM, including 18 females and 6 males, between March 2023 and October 2023. Patients were randomly assigned into one of two groups: the control group that received the SRP alone and the intervention group receiving SRP with ozone gel as adjunctive treatment (SRP + Ozone). Participants were recruited from the outpatient clinic, Department of Oral Medicine and Periodontology, Faculty of Dentistry, Cairo University. Patients were screened until the targeted sample size was achieved, which was adjusted for possible dropouts. The trial protocol was registered on www.clinicaltrials.gov protocol registration and results system on September 9, 2022 (with an identifier ID: NCT05538078). The Ethics Committee of the Faculty of Dentistry, Cairo University approved the research protocol and informed consent templates in July 2022, and consent was obtained from all individuals included in the study. The present study was conducted according to the Helsinki declaration principles of 1975, as revised in 2013 and reported according to CONSORT guidelines [18] (Fig. 1).

### Study population

The inclusion criteria comprised patients with stage III grade B periodontitis. Stage III periodontitis was defined by having interdental CAL ≥ 5 mm, radiographic bone loss extending to the middle or apical third of the root, and presence of pocket depth (PD) ≥ 6 mm. Whereas, grade B periodontitis was characterized by a moderate rate of disease progression with < 2 mm radiographic bone loss over 5 years [6]. The study included patients with controlled type II DM (HbA1C < 7), as outlined by Mori et al. [19], aged > 18 years, who agreed to a 6-month follow-up period and provided informed consent. Patients with prosthetic crowns, extensive restorations, teeth mobility greater than grade I, periodontal therapy within the last 12 months, underwent surgical therapy or orthodontic treatment, ongoing drug therapy that might have an impact on the clinical signs and symptoms of periodontitis, and antibiotics or anti-inflammatory drugs administration one month prior to the procedure and until the end of 6 months of follow-up, were excluded. Furthermore, smokers, pregnant females, or people with any known allergies were excluded from the study.

### Sample size, randomization, and blinding

The sample size was calculated using data from a prior study [20]**.** The response within each subject group was normally distributed, with a standard deviation (SD) of 1.09 mm. Alpha was set at 0.05, and the power was set at 0.8. The effect size for Student’s t-test (d) was 1.5. Therefore, the number of patients required in each group is 9. Then, we increased the sample size to 12 subjects per group to compensate for an anticipated dropout rate of 15% after 3 months, with a total of 24 participants. The sample size was calculated using G-Power software Version 3.1.2 (G*Power: statistical power analysis program, Version 3.1., Heinrich-Heine-Universität, Düsseldorf, Germany). A computer-generated randomization list was carried out by a physician uninvolved in recruitment to ensure concealment. The allocation sequence was then revealed after completing scaling and root planning. Subsequently, the allocation sequence was disclosed following scaling and root planning completion. Participants were assigned to either the SRP+Ozone (test group) or the SRP group (control group). The outcome assessors (NS & AM) and the biostatistician were blinded; however, blinding was not feasible for the principal investigator or the participants, as those in the intervention group received a specific treatment.

### Clinical outcomes

Clinical attachment level (CAL) was assessed as the primary outcome, defined as the distance from the cementoenamel junction (CEJ) to the bottom of the gingival sulcus. Probing pocket depth (PPD) was measured as the distance from the gingival margin to the base of the pocket. Measurements were taken at six sites: mesio-buccal, buccal, disto-buccal, mesio-lingual, lingual, and disto-lingual, using a UNC 15 periodontal probe (Nordent Manufacturing Inc., IL, USA). The full mouth plaque score was defined as the percentage of sites with plaque [21], and the full mouth bleeding score was defined as the percentage of sites showing bleeding on probing) [22]**.** These scores were assessed at four sites for each tooth: mesial, buccal, distal, and lingual. All clinical outcomes were measured at baseline, 3, and 6 months.

### Radiographic outcomes

**Fig. 2:**
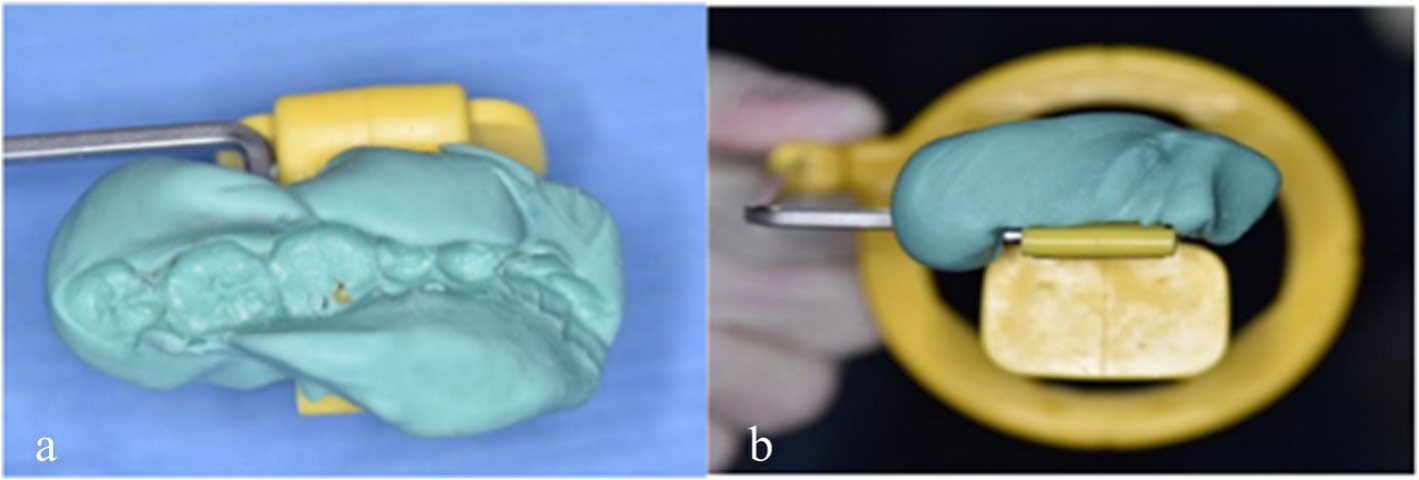
Customized bite registration stent with parallel x-ray holder for a standardized periapical radiograph

Radiographic linear defect depth (RLDD) was measured as the depth of bone defect from the cementoenamel junction (CEJ) to the base of defect at baseline and 6 months postoperatively to assess the extent of bone fill [23, 44]. A customized bite-plate registration was fabricated for each participant to standardize the position of radiographs using putty silicon (Zhermack Zetaplus C-Silicone Kit Italy). Radiography of patients was conducted utilizing the parallel-angle standardized radiographs technique, characterized by parameters of 60 kVp, 8 mA, a focal spot of 0.7 mm, and exposure times ranging from 0.02 to 3.2 s using periapical X-ray image plate (the Durr Dental Sensor, size 2, Germany) with the XCP® x-ray film holding system (Dentsply Rinn, USA) **(**Fig. 2**).**

**Fig. 3:**
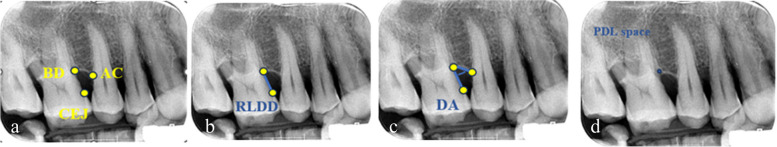
Radiographical measurements. a Identifying reference points: cementoenamel junction (CEJ), bottom defect (BD), and alveolar crest (AC). b Determine radiographic linear defect depth (RLDD)(CEJ-BD). c Determine the defect angle (DA) and join CEJ, BD, and lateral border of the defect. d periodontal ligament space (PDL space) measurement

Two reference points relevant to each defect site, namely, CEJ and BD (the bottom of the defect), and one reference line parallel to the treated teeth root surface. It was important to consider the distance between the CEJ and the most apical point of bone defects. This distance, known as the CEJ-BD distance [23], was then measured by distance ruler tool in millimeters using the ImageJ analysis software program (ImageJ Software, Research Services Branch, NIH, Bethesda, Maryland, USA) for the computer-assisted radiographic analysis [24], as shown in Fig. 3**.**

The radiographical defect angle at baseline was determined linearly as the angle between two lines: the first line connecting CEJ to BD along the root surface. The second line represents the lateral defect border, defined as the distance from the most apical part of the defect to the coronal part of the alveolar bone crest in contact with adjacent teeth, as described by a previous study [25]. The defect angle was measured using the ImageJ analysis software angle measurement tool. In addition, the periodontal ligament space width was radiographically assessed in the selected teeth. The measurement of periodontal ligament space widening was conducted using a 3 × magnification tool from the software. A horizontal line was drawn along the lateral surface of the selected teeth, specifically at the interface between the most coronal aspect of the alveolar bone and the tooth root, as well as the adjacent bone. Finally, the measurements of this distance were conducted in mm using the software’s linear measurement tool [26]. Alternatively, in instances of invisible PDL space, two vertical points were positioned along the root surface, and the average width was calculated [27] or recorded the minimum width [28], as shown in Fig. 3.

### Preoperative phase

Baseline clinical intraoral photographs were taken to document treatment progress, and additional photographs were taken at 3 and 6 months postoperatively to assess the different treatment outcomes. Hemoglobin A1c (HbA1c) levels were measured in both groups, and the results were expressed as percentages [29]. The HbA1c levels were determined at the baseline and the 6-month follow-up. Periodontal examination and full periodontal charts were recorded for each participant at baseline and 3- and 6-month follow-up.

### Operative phase

Patients in both groups received comprehensive nonsurgical periodontal therapy in one visit, including SRP, to remove the hard and soft deposits from both dental arches. Subgingival debridement was performed under local anesthesia using professional instrumentation by both ultrasonic scalers (Woodpecker UDS-P with LED, China) and hand instruments such as Gracey curettes, after-five Gracy curettes and universal curettes (Hu-Friedy, Chicago, IL, USA). In addition, rubber pads and prophylaxis paste (Alpha-Pro® Prophylaxis Paste, USA) were used for the polishing procedure. The root surface was meticulously debrided to achieve a smooth and firm consistency. Subsequently, oral hygiene instructions and biofilm control measures were implemented at the three-month follow-up, including supragingival plaque removal and reinstruction of oral hygiene procedures.

**Fig. 4:**
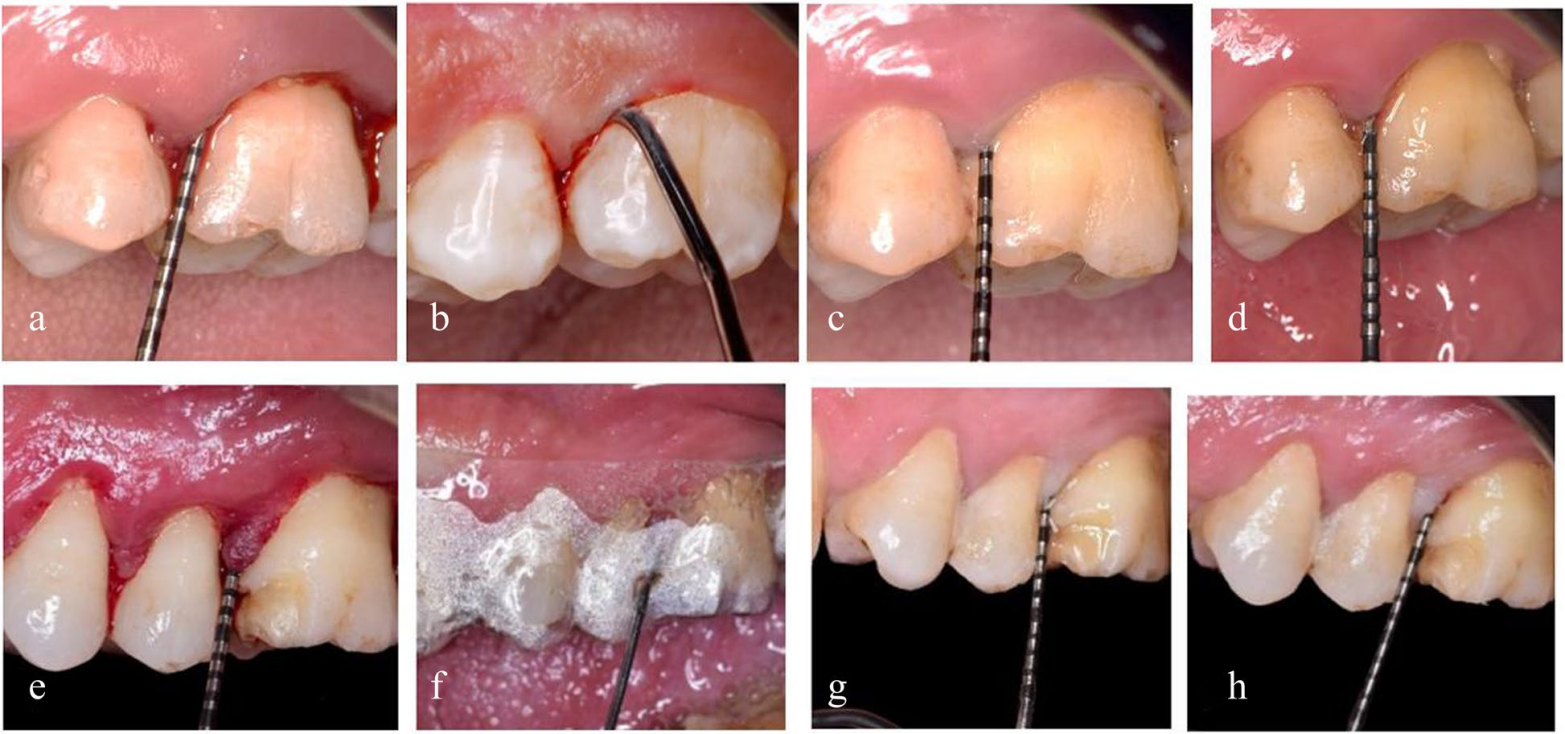
Clinical steps in representative cases of the control **(a-d)** and intervention **(e–h)** groups. Scaling and root planning (SRP) alone in Control group and intervention group utilize Ozone gel following SRP. Control group: **a** 6 mm probing pocket depth (PPD) with a 5 mm clinical attachment level (CAL) at baseline. **b** SRP using Gracey curette. **c** 3 mm PPD with 3 mm CAL at 3 months. **d** 3 mm PPD with 3 mm CAL and at 6 months. Intervention group: **e** 6 mm (PPD) with 4 mm (CAL) at baseline. **f** application of Ozone gel into the pocket following SRP using a 3 ml disposable syringe and acrylic stent. **g** 3 mm PPD with 3 mm CAL at 3 months. **h** 2 mm PPD with 2 mm CAL at 6 months

After the SRP procedure, patients were instructed to record their sensitivity level and perceived discomfort score for each tooth using a VAS at baseline and again seven days post-treatment. The hypersensitivity assessment utilized an air-blast apparatus directed at the root surface, with the syringe positioned perpendicularly to the surface, as illustrated by Tammaro et al. [30].

Participants in control sites were treated with SRP, including supra/subgingival debridement only. In contrast, subjects in the intervention group received SRP and ozone gel application (Geli O3, Bio-ozonized olive oil (20 mEq O_2_/Kg), Bioemmei Srl, 36100 Vicenza, Italy) administered via a 3 ml disposable plastic syringe and a blunt 23-gauge needle. Following the completion of SRP, the treated teeth were carefully isolated with cotton rolls and thoroughly dried. A previously customized transparent acrylic stent (Atoms, Thermoforming plastic, Germany) for each patient was placed on the arch, and the syringe nozzle was inserted through a round opening made in the acrylic stent, to ensure the gel flowed into the pocket. A range of 1-1.5ml of Ozone gel was then carefully applied sub-gingivally and interproximally until excess gel was visible along the gingival margin. The excess gel was removed with a cotton roll, as demonstrated in Fig .4**.**

### Postoperative care and Follow-up visits

Patients were instructed to wear the stent filled with ozone gel for a minimum of 30 minutes and to refrain from eating, drinking, rinsing, or chewing sticky food. No antiplaque agents, systemic antibiotics, or anti-inflammatory drugs were prescribed. Furthermore, they were prevented from using dental cleaning aids that may interfere with the ozone gel application site for at least 2 days after treatment. Visits were arranged for three and six months following the operation. Periodontal parameters were measured at 3 and 6 months postoperatively. Periapical radiographs were obtained 6 months following the baseline visit.

### Statistical analysis

Numerical data were explored for normality by checking the distribution of data and using tests of normality (Kolmogorov–Smirnov and Shapiro–Wilk tests). All data showed non-normal (non-parametric) distribution while age, CAL, PD and blood glucose level (HBA1c) data showed normal (parametric) distribution. Data were presented as median, range, mean and standard deviation (SD) values. For non-parametric data, Mann–Whitney U test was used to compare between the two groups. Friedman’s test and Wilcoxon signed-rank test were used to study the changes by time within each group. Dunn’s test was used for pair-wise comparisons when Friedman’s test is significant. Wilcoxon signed-rank test was used to study the changes within each group regarding VAS scores, RLDD, DA and PDL space. For parametric data, repeated measures ANOVA test was used to compare between the two groups as well as to study the changes by time within each group. Bonferroni’s post-hoc test was used for pair-wise comparisons when ANOVA test is significant. Student’s t-test was used to compare between mean age values in the two groups. Qualitative data were presented as frequencies and percentages. Fisher’s Exact test were used to compare between the two groups. The significance level was set at P ≤ 0.05. Statistical analysis was performed with IBM SPSS Statistics for Windows, Version 23.0. Armonk, NY: IBM Corp.

## Results

The baseline characteristics of the 24 participants, divided into two groups, are presented in Table 1. No significant differences were observed in baseline variables between the two groups after allocation [*p* > 0.05].

**Table 1:** Baseline differences at the time of allocation in two groups (*n* = 12 in each group)

Variables	SRP alone	SRP + Ozone	p-value
Gender			
Females	11	7	0.155
Male	1	5	
Mean age (years)	46.7 ± 4.7	44.3 ± 5.5	0.278

Fisher’s Exact test for gender and Student’s t-test for age

*SRP* scaling root planning

Table 2 presents the intra-group mean differences in periodontal measurements from baseline (0) to the subsequent post-intervention follow-up period for participants in each group.

**Table 2:** Post-intervention changes in clinical variables at different follow-up visits

Variables	SRP alone	SRP + OzoneQ	p-value
			(Between groups)
	(*n* = 12)	(*n* = 12)	
Mean CAL (0 m)	6.75 ± 1.22^A^	7.5 ± 1.78^A^	0.141
Mean CAL (3 m)	4.42 ± 1.38^B^	4.2 ± 1.87 ^B^	0.833
Mean CAL (6 m)	3.7 ± 1.06 ^C^	4.11 ± 1.76 ^B^	0.541
p-value (Within group)	< 0.001*	< 0.001*	
Mean CAL change (3 m)	2.33 ± 1.08	3.6 ± 1.71	0.055
Mean CAL change (6 m)	3.2 ± 1.14	3.89 ± 1.76	0.287
Mean PPD (0 m)	6.1 ± 0.32^A^	6.44 ± 0.53^A^	0.098
Mean PPD (3 m)	3.8 ± 1.23^B^	3.11 ± 0.78 ^B^	0.169
Mean PPD (6 m)	3.1 ± 1.1^C^	3.11 ± 0.78^B^	0.980
p-value (Within group)	< 0.001*	< 0.001*	
Mean PPD reduction (3 m)	2.25 ± 1.29	3.3 ± 0.67	0.045*
Mean PPD reduction (6 m)	3 ± 1.33	3.33 ± 0.71	0.256
Mean BOP% (0 m)	58.5 ± 8.7 ^A^	61.2 ± 13.4^A^	0.643
Mean BOP% (3 m)	23.8 ± 7.8^B^	22.6 ± 11.2 ^B^	0.869
Mean BOP% (6 m)	10.6 ± 6.8 ^C^	9.3 ± 7.6 ^C^	0.869
p-value (Within group)	< 0.001*	< 0.001*	
Mean PI % (0 m)	70.2 ± 14.4 ^A^	72.5 ± 15.8 ^A^	0.684
Mean PI % (3 m)	32 ± 6.2 ^B^	32.4 ± 9.1 ^B^	0.550
Mean PI % (6 m)	16.2 ± 6.6 ^C^	19 ± 8.8 ^C^	0.485
p-value (Within group)	< 0.001*	< 0.001*	
Mean VAS (0 m)	5.08 ± 1.16	4.75 ± 1.22	0.445
Mean VAS (1 week)	2.92 ± 1.62	1.42 ± 1	0.017*
p-value (Within group)	0.003*	0.002*	
Mean HbA1c% (0 m)	6.81 ± 0.13	6.79 ± 0.14	0.733
Mean HbA1c% (6 m)	6.56 ± 0.2	6.52 ± 0.16	0.656
p-value (Within group)	< 0.001*	< 0.001*	

Repeated measures ANOVA followed by Bonferroni’s post-hoc test for CAL, PD and HbA1c (Between and within group comparisons), Mann–Whitney U test for comparison between groups regarding CAL change, PPD reduction, BOP%, PI% and VAS, Friedman’s test followed by Dunn’s test for comparisons within group regarding CAL change, PPD reduction, BOP%, PI% and VAS, Wilcoxon signed-rank test for the changes within each group regarding VAS scores

*SRP* scaling root planning; 0 m = baseline, 1 m = 1-month, 3 m = 3-months, 6 m = 6-months

^*^Significant at P ≤ 0.05. Different superscripts in each column represent changes within each group

In both the intervention and control groups, there were significant mean reductions in all glycemic and periodontal variables [*p* < 0.05]. The radiographical variables in the SRP + Ozone group exhibited a significant decrease in RLDD and PDL space, as well as a significant increase in DA [*p* < 0.05]. Conversely, the SRP group did not demonstrate a significant change [*p* > 0.05], as illustrated in Table 3**.**

**Table 3:** Comparison between radiographic variables at different follow-up visits as well as the changes within each group

Variables	SRP alone	SRP + Ozone	p-value
	(*n* = 12)	(*n* = 12)	(Between groups)
Mean RLDD (0 m)	3.63 ± 2.02	3.44 ± 1.25	0.795
Mean RLDD (6 m)	3.42 ± 1.6	2.66 ± 1.25	0.220
p-value	0.888	0.008*	
Mean DA (0 m)	68.3 ± 25.6	62.8 ± 17.7	0.563
Mean DA (6 m)	66.8 ± 24.1	70 ± 12.5	0.870
p-value	0.092	0.007*	
Mean PDL space (0 m)	0.92 ± 1.12	0.28 ± 0.07	0.221
Mean PDL space (6 m)	1 ± 1.24	0.2 ± 0.04	0.014*
p-value	0.593	0.008*	

Mann–Whitney U test for comparison between groups regarding RLDD, DA and PDL space, Wilcoxon signed-rank test for the changes within each group regarding all variables

SRP, scaling root planning; RLDD, radiographical linear defect depth; DA, defect angle; PDL, periodontal ligament space; 0 m = baseline, 1 m = 1-month, 3 m = 3-months, 6 m = 6-months

^*^ Significant at P ≤ 0.05

Furthermore, there was no significant difference change found in periodontal outcomes between the SRP and SRP + Ozone groups [*p* > 0.05]. However, the SRP + Ozone group exhibited a significant reduction in PD at 3 months [*p* = 0.045].

Moreover, in the SRP group, there was no statistically significant change in radiographical measurements after 6 months, while the SRP + Ozone group showed a statistically significant change in radiographical measurements. However, there was no significant difference in radiographical parameters between both groups at 6-month follow-up compared to baseline, as illustrated in **Table (3)**.

Furthermore, the regression model results indicated that none of the variables served as a significant CAL predictor after 6 months.

## Discussion

Periodontitis is widely recognized as a risk factor that can exacerbate glycemic control and increase the likelihood of developing diabetic complications, as demonstrated by Mealey et al. [7]**.** In addition, Lalla and Papapanou [31] demonstrated that DM affects the onset and progression of periodontitis by eliciting an excessive inflammatory response, hindering bone repair processes, and generating advanced glycation end products.

Nonsurgical periodontal therapy (NSPT) focuses on managing microbial periodontal infections by eliminating bacterial biofilms and toxic substances from root surfaces, significantly reducing inflammation and improving periodontal parameters [32]**.** Moreover, Baeza et al. [10], conducted a meta-analysis demonstrating that NSPT can improve metabolic control and reduce systemic inflammation in patients with T2DM, as indicated by lower serum levels of HbA1c and CRP, respectively. Complete elimination of microorganisms is challenging, resulting in persistent microbial activity. Consequently, the use of various antimicrobial agents as an adjunct to mechanical debridement is essential [33]**.**

There is a lack of data in the literature concerning the effect of ozone gel on the clinical parameters associated with periodontal inflammation in diabetic patients. To the best of the authors’ knowledge, this is the first randomized controlled clinical trial to evaluate the clinical and radiographical effect of subgingival application of ozone gel in periodontal pockets combined with SRP versus SRP alone in controlled diabetic patients with stage III grade B periodontitis.

Regarding CAL measures, the current trial reported a mean difference of (2.33 and 3.05 mm) in SRP group at 3 and 6 months, respectively. This CAL improvement is consistent with the findings of Soi et al. [34]. Furthermore, the mean change in CAL measurements for the SRP group at six months aligns with the findings of Kolte et al. [35]. Nevertheless, Jain et al., conducted a systematic review and found that the effect of SRP on CAL resulted in a non-significant reduction of 0.22 mm, which is less than the reduction observed in our study after 3 months of treatment [36]. Our findings regarding the SRP + Ozone group are consistent with those presented by Colombo et al. [33]. This could be attributed to the stimulating impact of ozone on fibroblasts, promoting the repair of connective tissue [33]. These findings are also supported by Ranjith et al. [17] and Scribante et al. [37]. Conversely, the present findings do not align with those of Tokede et al. [38]**,** who reported slight and non-significant improvement in CAL measurements following ozone therapy.

The pocket depth (PD) measurements indicated no statistically significant differences among the studied groups at baseline, after three months, or after six months, consistent with previous studies by Moraschini et al. [39] and Soi et al. [34]**.** The favorable outcomes of this study are linked to the implementation of a customized acrylic stent, utilized for at least 30 min to maintain the gel’s position, in contrast to the other study that allowed the gel to remain for a minimum of 2 min. The intergroup comparison in the study indicated a statistically significant improvement in PD reduction at 3 months (P ≤ 0.05). The results contradict those of Uraz et al. [40], who reported no statistically significant differences between the groups after the administration of ozone in gaseous form. This non-significant difference may be due to the instability of ozone in its gaseous state, which has a lifespan of approximately 30 to 40 min. Additionally, the oily form of ozone demonstrated the most effective clinical outcomes [41].

The current study found no significant difference in the mean plaque index (PI) between the SRP and SRP + Ozone groups. Both groups demonstrated a statistically significant reduction in BOP after three months and from three to six months, consistent with the findings of Al Habashneh, Alsalman, and Khader [42]**.** Conversely, Marconcini et al. [39] reported that diabetic patients receiving ozone therapy in conjunction with another antioxidant agent significantly reduced bleeding compared to those treated with chlorhexidine at 3 months. However, in their study, periodontal health worsened after six months, with all indices reverting to baseline values. Moreover, Kolte et al. [35] demonstrated a non-significant decrease in the bleeding index following NSPT after 6 months.

With regards to postoperative hypersensitivity considered as the subjective outcome, the current clinical trial demonstrated that patients receiving either SRP alone or SRP combined with Ozone gel experienced a significant reduction in dentinal hypersensitivity (DH) during the first week. However, pain scores were higher in the SRP group. This aligns with previous studies showing a reduction in VAS score from 3 to 6 weeks following the use of adjunctive gaseous ozone [43]. However, discomfort was most pronounced on the first day postoperative and subsequently decreased. Both groups observed a statistically significant reduction in HbA1c measurements after six months. The current results align with the reviews conducted by Baeza et al. [10] and Marconcini et al. [39]**.**

The radiographic measurements related to RLDD, which assessed vertical bone gain in a coronal direction [44], revealed that SRP in diabetic patients led to a non-statistically significant reduction in bone defect depth from baseline to 6 months, with a mean change of (−0.21 ± 0.42 mm). This finding is consistent with Isidor et al. [45]**,** who reported no bone change after SRP. Moreover, Jeffcoat et al. [46] and Machtei et al. [47] both observed no variations in bone height with a lesser mean bone change (−0.14 ± 0.04) (0.16 ± 0.2 mm), respectively.

The analysis of the radiographical defect angle (DA), as recommended by Klein et al. [44], indicated a non-significant decrease in the SRP group, with a mean of 68.3 ± 25.6° at baseline and 66.8 ± 24.1° at 6 months. This finding was inconsistent with Nibali et al. [48]**,** who reported that debridement of the root surface with no adjunctive treatment was associated with a significant increase in defect angle with a mean of 44.1 ± 1.5° after 12 months compared to the baseline condition 37.4 ± 0.8°. This resulted in the complete resolution of the infrabony defect in some cases. Their superior results might be attributed to their long follow-up period and the good oral hygiene measures implemented by patients. This finding agrees with another retrospective study by Nibali et al*.*, [49] evaluating 35 periodontal bone defects treated with minimally invasive nonsurgical therapy (MINST). The results indicated a significant improvement in DA compared to the current study, with the average defect angle changing from 28.5° at baseline to 44.4° at 12 months (*p* < 0.001). Remarkably, the ozone group demonstrated a statistically significant increase in the DA from baseline to 6 months with means of 62.8° ± 17.7 and 70° ± 12.5, respectively. However, this remains significantly lower than the findings of Nibali et al*.* [49]**.**

The current randomized clinical trial indicated no significant differences in the periodontal ligament space (PDL) width in the SRP group at 6 months compared to baseline. By comparing the two studied groups, the SRP group exhibited a statistically significant increase in PDL space compared to the SRP + Ozone group. The findings align with those of Huth et al. [50]**,** who studied the influence of ozone on the host immune response and concluded that NF-κB activity in PDL tissue from the root surfaces of periodontally damaged teeth was inhibited following incubation with ozonated media. In another clinical study, Tayman et al. [27] demonstrated that the measurements conducted in their ex vivo study indicated PDL space widths typically range from 0.16 to 0.28 mm, aligning with the current study’s findings.

The present investigation encountered several limitations, including the limited number of articles in the literature addressing ozone therapy in periodontitis diabetic patients with similar clinical and radiological outcomes. Additionally, scares data were found concerning the use of VAS score system in evaluating the pain following subgingival debridement adjunctive with ozone therapy in diabetic patients, which posed a challenge in making comparisons between the reported outcomes of the present study and those found in the existing literature. Further research is required to clarify the therapeutic potential of ozone therapy in periodontal diseases, encompassing its mechanisms, optimal dosage, frequency, and application methods. Furthermore, radiographic analysis of bone density changes following scaling and root planning requires further elucidation. Modifying the paralleling film holder can standardize and facilitate serial radiograph comparison, thereby yielding valuable data.

## Conclusion

The current study concluded that adjunctive ozone therapy might have promising outcomes in the management of periodontitis in patients with type II DM. Ozone gel is suggested to be beneficial in periodontal therapy due to its simplicity, non-invasiveness, and lack of adverse effects. Scaling and root planing continues to be the gold standard for enhancing periodontal health and metabolic control in periodontitis patients with diabetes mellitus.

## Data Availability

The datasets used and/or analyzed during the current study are available from the corresponding author on reasonable request.

## Abbreviations

AC: Alveolar crest
BOP: Bleeding On Probing
BD: Bottom of defect
CEJ: Cemento-Enamel Junction
CAL: Clinical Attachment Level
DM: Diabetes Mellitus
HbA1c: Hemoglobin A1c
NSPT: Nonsurgical Periodontal Treatment
O3: Ozone
PD: Pocket Depth
PDL: Periodontal ligament space
PGE2: Prostaglandin E2
*PI*: Plaque index.
RLDD: Radiographic Linear Defect Depth
ROS: Reactive Oxygen Species
SRP: Scaling and root planing
VAS: Visual Analog Scale
